# How Can We Predict Treatment Outcome for Depression?

**DOI:** 10.1016/j.ebiom.2014.12.008

**Published:** 2014-12-18

**Authors:** Martin Walter, Anton Lord

**Affiliations:** a Leibniz Institute for Neurobiology, Magdeburg, Germany; b Clinical Affective Neuroimaging Laboratory, Magdeburg, Germany; c Department of Psychiatry and Psychotherapy, Otto von Guericke University, Magdeburg, Germany; d Center for Behavioral Brain Sciences (CBBS), 39106 Magdeburg, Germany

Major depressive disorder (MDD) is a highly pervasive disorder, affecting one in five people over the course of their life. Currently diagnosis is often based on the subjective opinion of health care professionals. The lack of an objective test increases the rate of mis- and delayed-diagnosis, often with people being later diagnosed with bipolar instead (Angst, 2007).

In an effort to identify the underlying mechanisms of MDD, recent studies have attempted to elicit disease specific properties of the cingulate cortex. Mayberg et al. (1997) who found hypometabolism in the rostal cingulate cortex predicted nonresponse to anti-depressants while cortical thinning in the dorsal anterior cingulate cortex is linked to clinical (Van Tol et al., 2013) and non-clinical cognitive trait markers in MDD (Li et al., 2014).

Clinical concepts of patient subgroups tried to relate different involvements of norepinephrine and serotonin transmitter systems to different clinical symptoms (Malhi et al., 2005). Furthermore functional connectivity between the anterior insula and pregenual anterior cingulate cortex was found to be modulated by glutamatergic levels (Horn et al., 2010) further suggesting a brain correlate of treatment selective subgroups in depression. Taken together, we have seen growing evidence of neurobiological alterations linked to depression, predicting treatment response to transmitter specific drugs.

Recently it was shown that MR-based classification is highly successful in identifying patients (Lord et al., 2012) using machine learning techniques and Redlich et al. (2014) showed that imaging based classification algorithms also work across countries and scanners. Thus the time seems ripe to test the performance on an imaging supported decision algorithm, which for the case of SSRI and SNRI so far remains largely intuitive.

Korgaonkar et al. (2015) now leverage data mining tools to identify biomarkers capable of predicting who will respond to selective serotonin reabsorption inhibitors (SSRIs) or serotonin–norepinephrine reuptake inhibitors (SNRIs). They use a technique known as decision trees to identify the treatment option most likely to succeed for an individual. This technique is particularly well adapted to clinical settings as it can not only predict the best treatment option but can also suggest further tests to help improve the accuracy of the prediction. Their successful example suggests that clinicians may in the future be able to inform treatment choice based on surrogate markers of brain integrity. This is an exciting advance as it offers the ability to tailor treatment strategies to the individual in an objective manner, improving the likelihood of successful treatment for individuals.

Such research represents an important proof of principle to overcome translational roadblocks which so far disconnect ample amount of evidence from non-invasive imaging studies and diagnostic clinical applications. Within the last 20 years, the availability of clinical MR systems has reached a critical mass where it becomes conceivable that patients can undergo simple structural or functional brain scans within the reach of their clinical environment. This infrastructural development is paralleled by mass accumulation of evidence on certain typical brain features which would underlie any neurobiologically derived clinical evidence.

Looking toward clinical applications, current and future large-scale imaging studies are uniquely equipped to advance knowledge into the classification of psychiatric patients. However, such an undertaking is typically beyond the scope of most individual research groups. Research in the field of psychiatric neuroimaging would benefit immeasurably from adopting open data sharing practices, similar to those that enabled rapid developments in genomics. Already we have seen progress in data sharing practices in clinical neuroimaging for other patient populations such as dementia (Mueller et al., 2005).

Similar databases are currently generated around the globe, including other diagnostic groups allowing quantitative diagnostic imaging tests for affective disorders that are robust, specific and sensitive will hopefully become a reality. Next to individualized treatment prediction which may not accumulate the same quantities of data, the next steps may further largely benefit from trans-diagnostic databases. In such a framework individual treatment responsivity may also be derived from localization of specific impairments within a multidimensional neurobiological framework irrespective of strict nosological categories.

## Disclosure

The authors declared no conflicts of interest.
